# A New Way of Identifying Biomarkers in Biomedical Basic-Research Studies

**DOI:** 10.1371/journal.pone.0035741

**Published:** 2012-05-11

**Authors:** Alexander Yassouridis, Tonia Ludwig, Axel Steiger, Friedrich Leisch

**Affiliations:** 1 RG Statistics, Max Planck Institute of Psychiatry, Munich, Germany; 2 RG Statistical Genetics, Max Planck Institute of Psychiatry, Munich, Germany; 3 RG Endocrinology of Sleep, Max Planck Institute of Psychiatry, Munich, Germany; 4 Institute for Applied Statistics and Computing, University of Natural Resources and Life Sciences, Vienna, Austria; Helmholtz Zentrum München/Ludwig-Maximilians-University Munich, Germany

## Abstract

A simple, nonparametric and distribution free method was developed for quick identification of the most meaningful biomarkers among a number of candidates in complex biological phenomena, especially in relatively small samples. This method is independent of rigid model forms or other link functions. It may be applied both to metric and non-metric data as well as to independent or matched parallel samples. With this method identification of the most relevant biomarkers is not based on inferential methods; therefore, its application does not require corrections of the level of significance, even in cases of thousands of variables. Hence, the introduced method is appropriate to analyze and evaluate data of complex investigations in clinical and pre-clinical basic research, such as gene or protein expressions, phenotype-genotype associations in case-control studies on the basis of thousands of genes and SNPs (single nucleotide polymorphism), search of prevalence in sleep EEG-Data, functional magnetic resonance imaging (fMRI) or others.

## Introduction

### Biomarker Searching: The statistical challenge of clinical and preclinical basic research

There is no doubt that the more facets of a complex phenomenon we can illuminate, the better we can explain the structure, mechanisms and alterations of this phenomenon. On the other hand, art of science is the ability to configure and describe a complex phenomenon with as few variables as possible but with a sufficient degree of detail, in order to make it comprehensible, plastic and operational. Therefore, when studying complex phenomena we must sail in both directions: firstly, to collect as much information as possible in order to explain the phenomena adequately and, secondly, to reduce the data abundance appropriately by identifying the most informative variables. Obviously, the restriction to the most informative data or to the most relevant factors that influence a complex phenomenon implies the renunciation of its complete and perfect explanation. At the same time, however, this restriction allows for a great gain in attractiveness and plasticity and makes such a complex phenomenon useful for practical simulations and further scientific investigations.

Higher biological organisms generally possess complex structures and are characterized by extreme intra- and inter-individual variability. This variability is not only due to thousands and thousands of genetic and epigenetic factors, but also to the fact that many of these factors vary with time, location and situation. Therefore, approaches to explain structures and behavioral mechanisms of biological organisms are of a difficult nature. Without exaggeration, we can ascertain, that in sciences, e.g. biology, psychiatry, physiology, econometrics etc., concerning behavioral investigations of such organisms, it is extremely difficult, if not impossible to find absolute and universally valid laws. Even in cases where the focus is on specific behavioral characteristics or alterations, we have to spend immense time and effort to explain them well and adequately. The required expense would be even larger if the topic of these investigations is the most intelligent and complicated biolog ical organism on earth, to wit the human being.

Being aware of the enormous complexity of higher biological organisms, scientists who investigate biological phenomena hope to identify those influential factors, that are able to characterize disturbed or extraordinary behavioral changes. The search for such relevant influential factors, known in the broader sense as ‘biomarkers’, is, therefore, an essential objective of almost all modern biological studies. This search, however, is not only from a biological but also from a statistical point of view a challenge per se.

It is well-known that the Achilles heel of the statistical inference is the hypothesis-testing via a statistical test. This is in principle a decision process (see [1]) of choosing between two possibilities (null versus alternative hypothesis) and the result of it like any decision may be either correct or incorrect. An incorrect decision in the statistical inference is associated with two risks known as Type I and II errors and denoted with the Greek letters 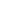 and 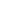, respectively. Type I error is the risk of rejecting the null hypothesis when it is true, and type II error is the risk of accepting the null hypothesis when it is false. Close connected to the statistical inference is also the test power or simply, power. Test power denotes the ability of a statistical test to reject a false null hypothesis, or in the case of a location test, its ability to detect true differences. From the definition of Type II error it follows that the test power is equal to 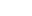.

But what are true differences and at which amount may they be declared relevant? In biomedical research one uses for the mean differences between two populations the term *biological difference*. The meaning and amount of biological differences are the basis for contention in many scientific conflicts. Here exist no unambiguous and clear answers. The amount of differences that have to be declared as relevant depends on many factors, e.g. on experimental and financial requirements, on the data types and scales, on sample heterogeneity, etc. Therefore, a great arbitrariness exists in the definition of relevant differences and just this arbitrariness extenuates the generalization of statistical inference to more complicated situations, for example, when more variables, different scales, more or multilevel factors exist.

Researchers behind basic-research investigations are not disposed to retrench scientific hypotheses concerning only one or a few variables. For scientists in this field all considered variables are a-priori important and, therefore, they relate their scientific assumptions to all of them, even in cases where some of the variables are declared as primary variables. Naturally, they hope to detect via statistical methods those few relevant variables or features that are interpretable, can help to explain well the variability of the investigated phenomenon and parallely could contribute to a reduction of noise effects and computational costs. A simple way in such situations is to postulate a complete or omnibus statistical hypothesis, taking into account all variables and selecting relevant variables by making statistical inference for each one of them on the basis of a procedure similar to multiple testing. Generally, statistical inference on a large number of variables is questionable, especially when the variables are measured in different scales and/or show dependencies (see e.g. [2]). Even under the assumption of independence and with the use of a uniform metric scale, statistical inference of multivariate data is liable to two risks: an inflation of the type I error and a deflation (weakness) of the overall power. To keep these risks small one has to strongly correct the level of significance and simultaneously operate with large sample sizes. The last task is usually not possible and strong corrections of the level of significance imply a very conservative detection procedure. This means, a lot of variables contributing to the explanation of an observed phenomenon may fail to be statistically significant and remain uncovered. Another way favored by some modern approaches (see more details in the discussion) make use of association models and pass thereon to variable selection and dimensional reduction. However, these approaches are also not completely free of statistical inference. When hypothesis testing is focused to a-priori defined subset of variables or factor levels, a noteworthy suggestion in the field of statistical inference is to refer to a partial null hypothesis and to partial or P-subset power (see e.g. [3] or [4]). But how can we know a-priori which e.g. genes are influenced by a disease? Therefore, the only promising solution in searching for biomarkers by complex biological investigations is a selective statistical inference applied to an adequate partial statistical hypothesis that concerns only of few of the most relevant (informative) variables detected in advance. The conception of an appropriate exploratory method that leads in an uncomplicated and reliable way to the identification of the most informative variables (biomarker candidates), independently of sample type, data nature, distribution requirements and so on, is the work and the challenge discussed below.

## Methods

### A new ‘distribution-free’ approach

We focus on the detection of relevant biomarkers in the two-samples situation, which implies an influential factor of two levels (two groups, two treatments, two time points, two experimental conditions, etc.). The generalization to more complicated situations and more factors is discussed in the end of this section. Following list summarizes the desired properties of the new method:

- Avoidance of statistical inference
- Possibility to also work in cases where there are strong dependencies between variables (collinearity, interactions)
- Applicable to different data types (metric, ordinal, categorical) and to different scales of measurement
- Applicable to different experimental designs

How does the new method work in order to accommodate these requirements?

### Measure of Relevance

**Figure 1:**
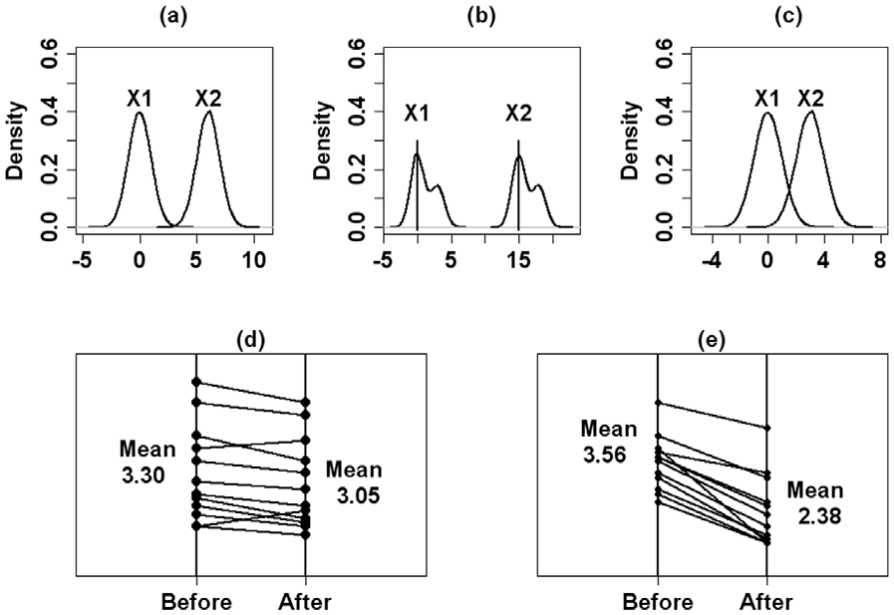
Example of different distributions of two random variables. Location and shape of two independent (1a, 1b, 1c) and two dependent random variables (1d and 1e).

Let us first address the question of which kind of information might be relevant in describing the difference of a variable in a two-sample problem and why. The graphs in figure (1) help us find the answer convincingly and objectively. Figures (1a), (1b) and (1c) represent three possibilities for the distributions of two continuous random variables, 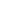 and 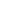, from two independent samples. The distributions of 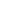 and 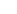 have identical shape in figures (1a) and (1c), however, the difference between their mean locations is larger in figure (1a). Because of the different degree of overlap a comparison of 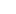 and 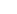 by means of a nonparametric test would yield a smaller p-value for the situation in figure (1a) than for that in figure (1c).

The distributions from figure (1a) differ from those in figure (1b) in shape as well as in location. The difference of the mean location in situation (1b) is larger than that one in situation (1a). In biomedical research one would speak of a larger biological difference in the case of (1b). If we consider, at the same time, the different variability degree of the variables and form the normalized biological difference, i.e. the ratio of observed difference and pooled standard deviation, the situations depicted in figure (1a) and (1b) might not be so different after all.

Figures (1d) and (1e) depict two situations for dependent samples. Obviously the mean differences for the two groups in figure (1e) are large compared to those in (1d). However, when applying a nonparametric test for dependent samples one will obtain significant results (small p-values) for both situations, although location and deviation of the two situations differ essentially from each other.

Summarizing the facts, from the above comparisons, we conclude that an adequate quantitative measure or function that would contain or reflect relevant information about sample differences in a variable, must maintain the following properties:

- it should incorporate a possible overlap in the distributions of the two samples
- it should express the biological difference on a uniform scale, at best on the interval 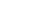

- it should incorporate the variance of the biological differences in an inverse relation in order to attenuate the effect of different metric scales on the biological difference
- it should incorporate the corresponding sample size in an inverse relation. This property will accommodate situations with missing values as well the fact that when sample sizes increase the probability of detecting small effect sizes as relevant should also increase

### The principle of the new method

In a two-groups situation let
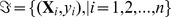
(1)be a sample of 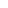 items, on each of which a vector 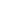 of 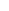 features 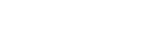 and a group-membership variable 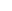, 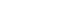 are considered.

In decision tree analysis, like CART (Classification And Regression Trees), Random Forest, AdaBoost, etc. (for an overview see [5]), which offer, in the explorative field, powerful ways to detect significant features and associations between them and the group variable, one generally searches for a function 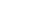 over the feature vectors 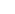, which fits the observed group variables 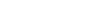 in the sample well. In mathematical notation it means that 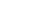 has to fulfill the condition:
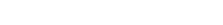
where 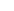 indicates the total prediction error.

The growth of the decision trees uses algorithms which determine by successive steps the best split-variable (feature) and its best splitting-value that could further improve prediction. Although greedy algorithms are very ingenious and fast, we can imagine how laborious decision tree analysis could be if 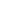 is very large 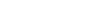. Moreover, when the two groups represent two dependent samples the fitting of 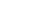 creates additional difficulties.

By our method we digress from the fitting principle used in the decision-tree analysis and follow another one working as follows: Instead of searching for a function 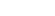 over the 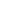 features that approximates the sample values 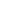, we first search for a function 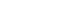, which for each feature (variable) 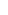 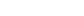 delivers important information about its differences between the two groups and then look for the features with the largest information values. We assign the wanted function 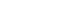, the name ‘measure of relevance’ (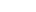). Therefore, the principle of the new method is not to search for the best classifiers, that enable optimal fitting in a sample, but rather to search for the best informators that give the best information over a sample's irregularities and distinctive characteristics. In a mathematical notation this principle could be outlined in three steps:

- definition of a function 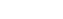 reflecting for all features 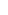 (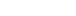) relevant information about the group or sample differences
- construction of an information chain by sorting the absolute values of 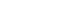 over 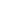

- definition of a selection- and evaluation criterion on the information chain

Dependencies between the features are in the first instance not of particular interest, because we are primarily focused on identifying features with information about the group difference and not on dependencies between features. Only after identifying features with relevant information (biomarker candidates) would it be advisable and interesting to further study dependencies between them and the other features.

Considering the aforementioned desired properties of a quantitative and informative measure towards sample differences, we find that a function, say 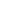, of form
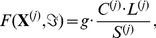
(2)where 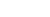 and 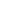 represent indicators for the distribution-overlap, the biological difference and the standard deviation of the pooled sample, contains the most useful information about the differences in feature 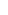 between the two samples. 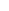 in the above formula indicates a weighting factor common to all features.

To meet the method's requirements (listed at the beginning of this section) the factors 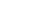 and 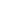, which have to be determined from the sampled data, must have on 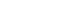 the same range values for all features 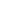 irrespective of the data type and sample design and guarantee comparisons in 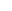 between features and/or between other samples. A good way to supply 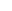 with these properties offers the rank- and U-transformations applied in succession. For each feature 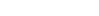 we first transform its values *in the whole sample* into ranks, say 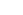, and then transform the corresponding ranks into the interval 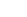 by using the formula 
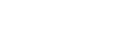
. In the end we obtain a new sample
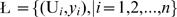
(3)with 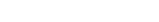.

Now let
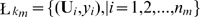
(4)denote the two subsamples of Ł with values 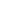 corresponding to the group 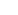 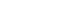. For dependent samples 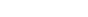.

Based on the samples Ł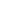, 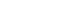 the factors 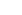, 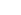 and 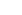 in (2) are defined as follows:
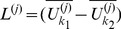
(5)

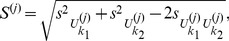
(6)where 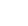, 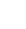 and 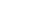 denote means, variances and covariance of the 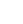 over the samples 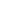, (m = 1,2; j = 1,2,…,p), respectively. For independent samples 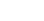 obviously equals 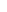.

In contrast to the definitions of 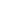 and 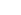, which are valid to any data type and any sample design, the definition and determination of 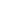 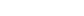 needs to differentiate between dependent and independent samples. By using the indicator 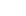 which equals 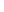 if z is true and 0 elsewhere, we define 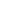 as follows:
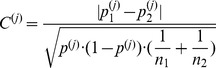
(7)where 
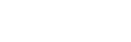
 and
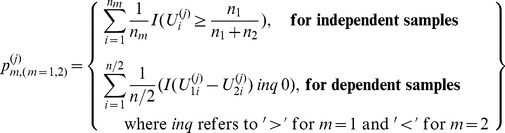
(8)


The expression in the right of (7) is identical to the statistic used for the parameter comparison of two binomial distributions when 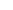 and 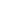 are large (see [6]). We use this formula for the calculation of 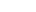 irrespective of sample size by differentiating between sample designs only. According to the underlying sample designs the 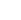 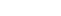 have to be determined differently.

Formula (8) means the following: For independent samples, 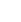 and 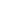 in the above formulas represent the proportions of the transformed data 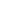 in the sample Ł that are larger than the quantile 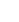 of the pooled sample, where 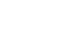. Since for the feature 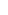, 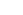 represent transformed values of consecutive ranks in the interval 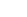 we can find after some algebra that 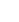 is equal to 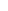 for any 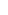. For 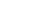, 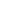 equals the median of the pooled sample, which is here 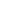.

For dependent samples 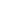 and 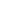 refer to the proportion of positive or negative differences, respectively. Ties will not be considered. Since for dependent samples 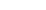, it is easy to conclude that 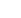 is here equal to 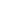 for any 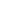.

Substituting the factors 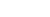 and 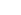 in formula (2) with the related expressions of (5)–(8) concerning to transformed data and setting for the weighting factor 
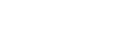
 we obtain for each feature 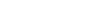 of the original sample 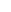 the corresponding 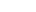. The complete and compact 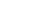-formula looks so:
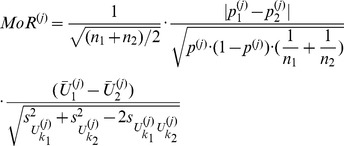
(9)


If we ignore the index 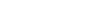 and focus on one feature (variable) only, the formula of the 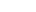 looks very simple. This formula is shown in Appendix S1 and can be used in calculation software programs for an easy determination of the measure of significance via algorithms.

Please note, while the 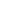
's are unbiased estimators of the difference between population means, 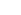
's are not unbiased estimators of the pooled standard deviation. The factors 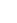 that represents the normalized biological differences are similar to the Hedges' 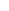 (see [7]). However, 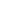 is an unbiased estimator of the effect size only by a multiplication with a certain factor. Besides Hedges' 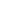 there are further estimators of effect size, for example, Cohen's 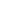 (see [8]) or Glass' 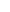 (see [9]) as well as other correlational effect-size indicators. Most of these effect-size indicators which include the factors 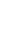 are characterized with small-sample biases. Nevertheless, provided that we are predominantly interested in an adequate measure of the information content and not in the development of a statistic and its distribution, the question of optimality and unbiased effect-size estimators does not affect the new method substantially.

### Notes

#### (a) Categorical data

Using the transformed sample values 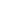 instead of the observed data values and determining therewith the unknown parameters in (9) we are able to calculate the measure of relevance for almost all common situations in a two sample-problem, namely for dependent or independent samples and for metric, ordinal and binary data as well, when binary data are coded by the numbers 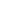 and 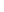. Exceptional situations may expose only nominal data with more than two outcomes. Of course, nominal data can be handled as ordinal categorical data too, but assigning numbers to their outcomes is an arbitrary act. However, for nominal data with more than two categories, this process should not be pursued. In reality we deal with multinomial distributions, and the transformation of their location and dispersion parameters into an one-dimensional parameter, like the measure of relevance, creates some difficulties. Provided that the sample sizes are sufficient, we suggest not considering nominal variables as predictors, but rather as influential or control variables.

#### (b) Avoiding ties in 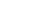


When operating with very small sample sizes and very large numbers of continuous variables, which for example is often the case in gene expression analyses, it is recommendable to use the original data to calculate 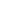 and 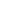 in formula 2 rather than using ranks and U-transformations. This is because for very small sample sizes the use of ranks and U-transformations will often produce normalized biological differences 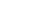 with equal values (ties) (It is easy to understand that the number of possible values of 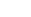 is equal to 
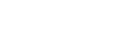
). Also, 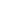 will be the maximal number of different 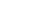 values, since the factor 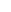 in 2 is transformation-invariant. In these cases, the discriminative power of 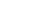 suffers and the only way to avoid this shortcoming is to operate with the original values. By doing so we take the risk of outlier effects which we accept, since clearing-up outliers is not particularly useful in very small samples.

### Selection Criteria

After receiving the 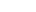
's for each feature 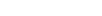 and constructing with their absolute values the information chain over 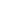 in the 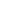 step, we then have to go to the last step of our method, which is to define a suitable selection and evaluation criterion in the information chain. We give below several selection criteria which help to determine the extent of relevant variables.

#### (a) Entropy

We are using entropy as a metric which describes the mean information content of a set of variables. It is a function which depends on the total number of variables in the considered set and the according relevance measure values. Changing the content of the set leads to a change in the entropy.

Let us assume that the set consists of 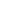 variables 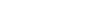 with 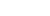 (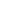 number of all considered variables), each of them featuring an information content of size 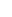. In our case, 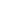 corresponds to 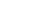 of the variable 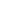. Assuming equal probability for all variables to be chosen as relevant, the entropy of the set of variables of size 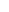 is given by
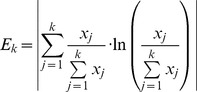
(10)


By sorting the absolute 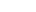-values of the variables 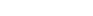 in decreasing order, we build 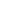 sets of variables, the first set, 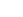 containing the variable 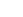 with the largest relevance value (first value of the information chain), the second set, 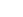 containing variables 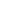 and 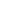 with the two largest relevance values and so on. For each of the sets we determine the entropy amount 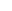. This value grows by adding more and more variables to the set. We stop adding variables to the set as soon as the change of entropy becomes negligible.

The following formula measures the adjusted change of entropy when adding the variable with the next smallest relevance value.
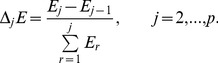
(11)


Therefore, when the condition 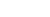, where 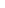 indicates a very small number, is for the first time fulfilled by the index 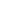, all variables in the information chain with index less than 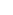 should be selected as informative.

#### (b) Permutation distribution

Another way to identify the number of relevant variables could be created by a permutation procedure.

For this purpose the group variable is permuted to derive a random group assignment among the observations. Thereby attention has to be paid that the original sample sizes remain stable for all permutations. Based on any new random grouping, we conduct the calculation of the relevance measure for all considered variables. The permutation procedure and calculation of the 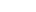 under random group assignment is repeated 
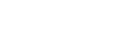
 times. By exhausting all possible permutations, we obtain for each variable 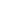 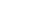-values. We then determine for each variable among its 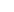 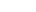-values a 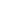-interval containing its 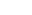 greatest 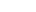-values, where 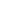 may be equal to a corrected or uncorrected 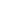, according to scientist's choice. Variables whose observed 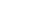-value are within the 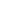-intervals should be declared as relevant. We made us aware of that for the selection of informative variables by means of permutations we do not implicitly want the information chain. However, to avoid too many calculations with the permutation method, it is advisable to focus only on variables that are at the first fragment of the information chain when 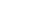
's are sorted in decreasing order.

#### (c) Cut-off criteria: Subjective cut-off criteria

By this approach, we are considering the following cut-off criteria:

- Sort the absolute 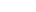-values of the variables 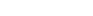 in decreasing order. 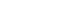 denote the corresponding ordered variables. Choose the first variables 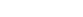 whose corresponding absolute 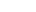-values 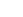 fulfill for the first time the condition 
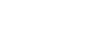
, where 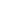 indicates a number less than 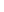. It is required that the information of the first 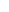 selected variable must be at least equal to a predefined fraction 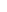 of the information supplied by all features.
- Choose a constant less than 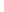, e.g. 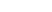, that will represent a proportion of the total number of desirable informative variables and choose the 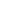 variables with the largest absolute 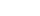.

#### (d) Cut-off criteria: Sample-related objective cut-off criterion

- Choose all variables with 
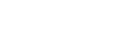
,

where 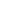 is a weighting factor equal to 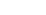 or 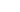 for small, moderate or large effects (see e.g. [8]). It is noteworthy that small, moderate or large effects should be desirable with large, moderate or small sample sizes corresponding to less than 30, between 30 and 60 or more than 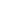 observations in both samples, respectively. Since for large samples the factor 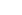 tends to increase (because its denominator decreases), the weighting factor 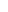 acts as a pedant and braking factor to the 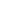
's. The number 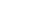 is indeed a product of simulations, but it can also be derived after some consideration. Because the factor 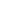 looks like to a Z-statistic, we expect that for large samples it will also have similar properties to this statistic. The 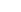
- and 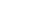-quantiles of the standard normal distribution give together about 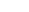. Compared to the entropy criterion the cut-off criteria are easy to conduct.

An interesting question regarding the stop-criteria is how to proceed with thse criteria as an optimal way to detect relevant biomarkers? After creating the information chain with the relevance measures of the considered variables it is recommended to first deal with an objective criterion that facilitates the decision of whether the information chain indeed contains informative variables or not. For this purpose, a good objective criterion is the sample-related objective cut-off criterion mentioned above. When the maximal absolute 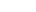-value in the peak of the information chain is less than 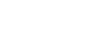, then the absolute 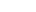-values of all other variables will also be less than these criterion-barrier. This implies that none of the investigated variables are informative. In such a case we do not want to make any further effort for the detection of biomarkers. However, if we find variables along the information chain with absolute 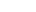-values greater than the corresponding threshold, then the search for biomarkers should be continued as follows:

If the number of variables whose relevance measure is greater than the criterion-barrier is too large then it is advisable to further use some subjective cut-off criterion to reduce the selection of the informative variables into a desirable amount. Again: an objective stop criterion should not be considered the ultimate ratio for selecting the most relevant biomarker candidates. In many situations we have to align the amount of the relevant biomarkers to practicability requirements and not to those of the stop criteria, independently of how objective the latter are. When, for example, in investigating allele frequency-distribution for 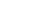 SNPs (single nucleotide polymorphism) between two sample populations we find, by a selection criterion, 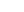 or more relevant SNPs, it would be impractical to consider all these SNPs as biomarker candidates for further analysis, especially not in cases with comparatively small sample sizes.

### Simulation studies

Provided that the measure of relevance represents a new metric scale it is absolutely essential to proof whether, and to which extent, it satisfies the three fundamental properties objectivity, reliability and validity. To examine these properties we conducted extensive simulation studies:

Since objectivity of a measure or a metric scale is its ability to reflect the information contained in the measured objects, we first simulated situations where 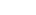 variables among which 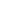 are informative and the rest non-informative have to be compared between two samples. We consider for the 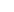 variables three degrees of information content: high, moderate and negligible. High informative variables are generally variables whose absolute amount of biological differences between two samples is higher than their pooled standard deviation, variables also with a normalized biological deviation larger than 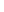. Variables with normalized biological differences near 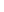 are semi-informative and those with normalized biological differences close to 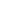 non-informative. However, as already mentioned, the normalized biological differences alone may not describe the relevance of the variables. One has to also consider the sample sizes to better analyze the degree of relevance. Therefore, we performed the simulation runs with different sample sizes. All variables (informative or not) are derived from the same distribution, but with different parameters, in order to assign them the desirable degree of information. If the 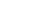 enables the identification of informative and non-informative variables correctly, independent of data structure, sample size and sample design, then the objectivity of the measure of relevance is highly guaranteed.

Reliability is more or less the answer to the question of how good a measure or metric scale can bear up against the practical test. In some scientific disciplines, where scales concern subjective assessments, reliability is often evaluated with agreement-coefficients of interrater or repetitions or intrarater scores. However, our measure of relevance concerns objective data, therefore, we need different instruments to test reliability. An appropriate method to proof reliability of the measure of relevance is the evaluation of its ability to identify relevant biomarkers in different situations (when the two samples derive for example from different distributions). To do so we simulated three different two-sample situations with 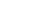 variables derived from a normal, uniform or a bimodal distribution, respectively. Also, 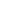 of the 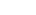 variables have high, moderate or negligible information content.

The question of whether the informative variables are indeed informative is verified by the validity test. To test the validity of 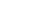 we compared the results from established statistical methods (see below) with the measure of relevance. These methods were a) the Random-Forest approach working on the explorative level and b) the multiple-testing procedure based on non-parametric tests (U-tests, Wilcoxon-tests or Sign-tests) working on the confirmatory level.

## Results

### (a) Objectivity

Table 1 gives a survey of the simulation results and delivers a better and deeper insight into the objectivity of the measure of relevance. When the ten exceptional variables are highly informative the simulation runs delivered, for both sample designs and both data structures (metric or binary), very good to excellent identification rates 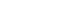, irrespective of sample sizes. This means, that the measure of relevance is capable of identifying almost exactly the 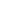 selected, very informative variables. Therefore, the measure of relevance shows a high sensitivity. When the 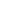 considered variables were semi-informative and have a normalized biological difference near 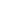 we can not expect that all of them will prove as informative in the simulation runs. Depending on the sample sizes, the part of the 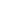 variables that can be identified as relevant in view of information content varies from moderate 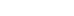 for small samples to high 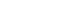 for large sample sizes. Because we are not able to know a-priori which of the 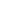 semi-informative variables are indeed informative, in each case we applied an appropriate non-parametric test. Those variables among them with p-values less than the adjusted 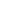, 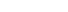 are considered informative. Interestingly, we found even for small sample sizes (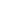 to 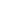) a good compliance (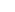 to 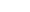) between 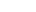 and the results of the non-parametric tests. For large sample sizes the compliance tends to be excellent even for semi-informative variables (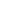 to 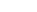). We also pursued the question of whether the part of the 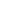 semi-informative variables that was not recognized as informative belongs indeed to the non-informative variables (specificity property). The specificity values were excellent (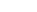) in all cases, i.e. irrespective of samples sizes, sample designs and data structure. Finally, when the 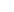 selected variables were like the rest of the non-informative variables, neither the measure of relevance nor the nonparametric tests detected any informative variables among them. This can be confirmed by the high specificity values (about 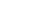), that remain high for any sample size and design. Overall, the objectivity property of the measure of relevance is acceptable.

**Table 1:** Objectivity of the measure of relevance.

		Independent Samples				Dependent Samples			
*Information content of variables (metric data)*		Prop. of inf. var. [%]	Sensitivity	Specificity	Sample Sizes ( 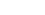 )	Prop. of inf. var. [%]	Sensitivity	Specificity	Sample Sizes ( 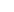 = 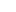 )
**a) very informative variables**									
abs. diff. in means (adm):	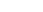	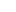 a; 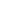 b	0.99	1.00	12; 10	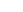 a; 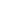 b	1.00	1.00	11
norm. biol. diff. (nbd):	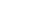	100; 100	1.00	1.00	15; 15	100; 100	1.00	1.00	15
		100; 100	1.00	1.00	30; 30	100; 100	1.00	1.00	30
**b) semi-informative variables**									
abs. diff. in means (adm):		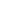 a; 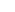 b	0.95	1.00	12; 10	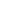 a; 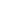 b	0.94	1.00	11
norm. biol. diff. (nbd):	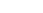	71; 73	0.98	1.00	15; 15	65; 66	0.97	1.00	15
		99; 99	1.00	1.00	30; 30	95; 97	1.00	1.00	30
**c) non-informative variables**									
abs. diff. in means (adm):	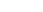	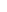 a; 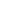 b	0.00	1.00	12; 10	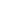 a; 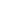 b	0.03	1.00	11
norm. biol. diff. (nbd):	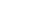	0; 0	0.00	1.00	15; 15	0.2; 0.3	0.04	1.00	15
		0; 0	0.00	1.00	30; 30	0.0; 0.0	0.09	1.00	30

Objectivity of the measure of relevance for the two sample-problem expressed in sensitivity and specificity indexes. Three two-sample situations were simulated. In each of them 1,000 variables among which 10 were very informative (normalized biological difference (NBD)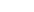), semi-informative (NBD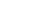) or non-informative (NBD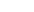), respectively. With 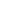 simulation runs the sensitivity (% correctly identified informative variables) and specificity (% correctly identified non-informative variables) were determined. Specificity is excellent and with increased normalized biological differences and sample sizes also the sensitivity indexes go up to excellent levels 100% (for more details see text).

a Rates (in %) of variables detected as relevant by the 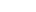.

b Rates (in %) of variables detected as significant by applying appropriate nonparametric tests.

**Figure 2:**
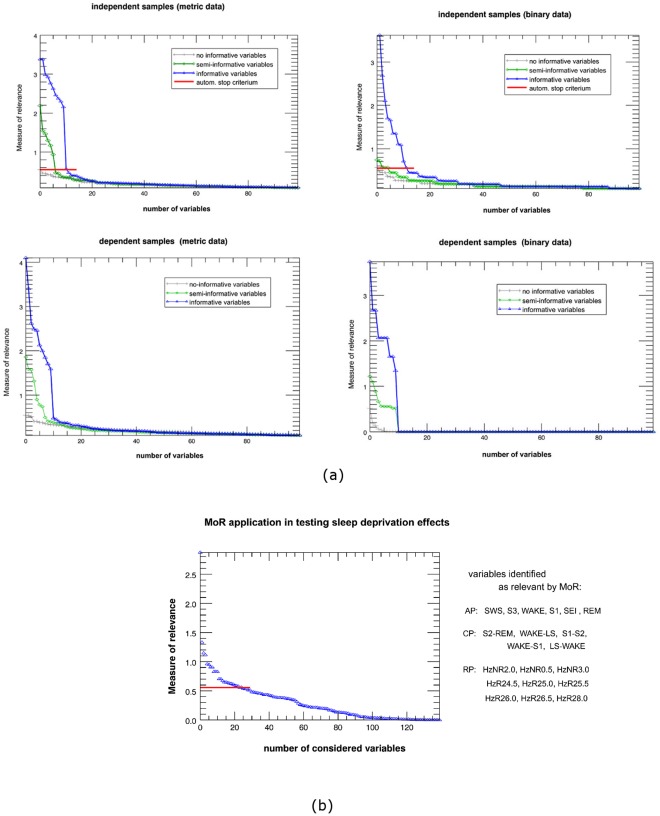
Measure of relevance for simulated and authentic data. *(a)* Courses of the best 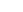 MoR-values by simulating a two-sample problem with 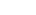 variables of different data types and sample designs. For each variable and each sample 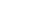 pseudo random numbers following a certain distribution (see more details in text) were drawn. The three lines correspond to situations with 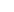 high-informative, semi-informative or non-informative variables among the considered 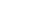 variables, respectively; all other 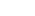 variables were non-informative. Irrespective of data type and sample design the 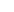 informative variables (blue lines) were correctly identified with 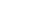. As expected the semi-informative variables showed lower 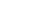-values and were only partially detected as relevant. *(b)* Application of the MoR approach to sleep EEG data in order to investigate the effect of sleep deprivation on sleep behavior. 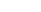 subjects were examined in two nights, before and after sleep deprivation. Dataset consists of different data types (metric, ordinal, binary etc.). The MoR-values of the parameters over the solid (red) line are all greater than the sample-related cut-off criterion 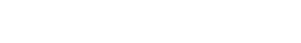.

Figure (2a) visualizes the objectivity of the measure of relevance for simulated metric and binary data of independent and dependent samples by using small samples (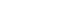). The data generation in simulations with samples with binary data structure was based on Bernoulli-distributions 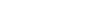, where 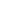 was selected in such way that the normalized biological differences equals about 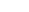, 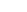 or 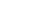. With the 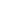 pairs 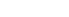, 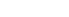 and 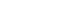 for the samples we generated pseudo-random numbers equal to 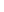 and 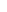 having the desired properties. Variables whose absolute 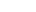 is placed at the beginning of the x-axis have the largest information content. For both sample designs and both data structures the ten variables with largest information content revealed very large 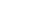-values in comparison to the other variables; therefore, they were placed at the beginning of the graph. Application of the sample-related objective cut-off criterion 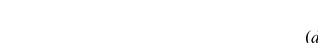 clearly revealed these informative variables (see horizontal red lines in the upper).

### (b) Reliability

As mentioned above, we have to simulate variables that are derived from different distributions for testing reliability. Next to the normal distribution, which was already used to test objectivity (see table 1), we chose for the variables two other distributions, namely, a uniform and a bimodal (mixed) distribution. To obtain the same normalized biological differences with these distributions as with the normal distribution used in the objectivity considerations, we chose suitable distribution parameters. For example, since the standard deviation of a uniform distribution U on 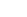 is equal to 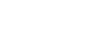, we have to draw pseudo-random numbers from a uniform distribution 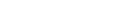 for one sample and a uniform distribution 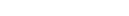 for the other in order to obtain 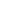 very informative variables with normalized biological difference of about 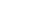.

Table 2 shows the reliability results from the simulation runs. For the 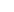 informative or semi-informative variables obeying the uniform distribution, we obtain results similar to that for the normal distribution (see table 1) for both sample designs. However, if the variables follow a bimodal distribution, the rate of detecting variables as informative is for small samples low, even in the case of variables with large informative content. This is not surprising. Variables following a bimodal distribution have large variances and yield poor mean estimations in small samples. Standardized mean differences based on these variables are, therefore, also affected by small samples. The 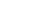 as well as the inferential tests are more effective in cases of large sample sizes because with large samples means of differences or effects can be assessed more accurately. Nevertheless, the sensitivity and specificity of 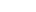 are large (from 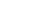 to 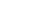), even in the case of variables with bimodal distribution. This indicates the excellent diagnostic capability of it in informative and not-informative situations.

**Table 2:** Reliability of the measure of relevance.

Independent Samples									
		Uniform distribution				Bimodal distribution			
*Information content of the variables*		Prop. of inf. var [%]	Sensitivity		Specificity	Prop. of inf. var [%]	Sensitivity	Specificity	Sample Sizes ( 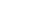 )
		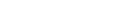				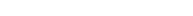			
		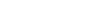				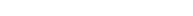			
**a) very informative variables**									
adm:	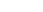	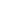 a; 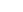 b		1.00	1.00	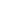 a; 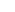 b	0.84	1.00	12; 10
nbd:	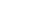	100; 100		1.00	1.00	70; 64	0.90	1.00	14; 16
		100; 100		1.00	1.00	97; 92	0.95	1.00	30; 30
		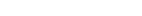				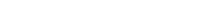			
		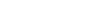				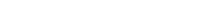			
**b) semi-informative variables**									
adm:	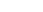	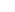 a; 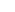 b		1.00	1.00	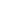 a; 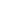 b	0.88	1.00	12; 10
nbd:	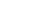	58; 68		1.00	1.00	60; 57	0.92	1.00	14; 16
		90; 96		1.00	1.00	92; 90	0.96	1.00	30; 30

Reliability of the measure of relevance for the two sample-problem expressed in sensitivity and specificity indexes. Two different distributions, uniform and bimodal, were used for evaluating reliability. Like the objectivity also the reliability property of the relevance measure goes up to excellent levels (sensitivity and specificity about 100%) with increased normalized biological differences and sample sizes.

a Mean rates (in %) of the variables detected as relevant or significant by 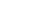.

b Mean rates (in %) of variables detected as relevant or significant by applying appropriate nonparametric tests.

### (c) Validity

The aim here is to show that the introduced method, based on 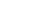, is at least as good as a classic inferential approach or a well-known exploratory approach. As already mentioned, we use the multiple testing and the Random Forest (see [10]) as comparative approaches. We use for both independent and dependent samples only metric data. For the simulation study, we performed 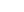 simulation runs in each of which 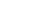 variables were generated as described above. To compare the selection quality of the 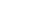 with the aforementioned methods, we used the sample-related objective cut-off criterion. Among the three methods applied to the data, the 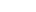-method and Student's t-Test without 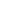-adjustment indicate excellent and almost identical sensitivity values for informative and semi-informative variables (see table 3). This observtion is independent of sample design and size. The Random Forest shows a weakness of sensitivity in the detection of informative and semi-informative variables, which can be explained with the appearance of strong pseudo-correlations between these variables and some of the 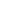 non-informative variables. The specificity values however, which vary from good to very good for all methods, show some discrepancies between the Random Forest method and the 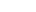 and multiple-testing method, especially for large sample sizes. However, this should not be interpreted as a weakness of the new method.

**Table 3:** Validity of new method compared to alternative methods.

	Independent Samples			Dependent Samples		
*Method*	Sensitivity	Specificity	Sample Sizes ( 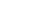 )	Sensitivity	Specificity	Sample Sizes ( 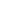 = 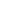 )
**a) very informative variables**						
New Method:	1.00	0.86	12; 10	1.00	0.82	11
T-Test without 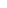 -adjustment:	1.00	0.95		1.00	0.95	
T-Test with 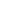 -adjustment:	0.90	1.00		0.90	1.00	
Random Forest:	0.30	1.00		0.50	1.00	
New Method:	1.00	0.78	14; 16	1.00	0.69	15
T-Test without 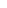 -adjustment:	1.00	0.95		1.00	0.95	
T-Test with 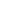 -adjustment:	1.00	1.00		1.00	1.00	
Random Forest:	0.20	1.00		0.20	1.00	
New Method:	1.00	0.56	30; 30	1.00	0.41	30
T-Test without 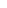 -adjustment:	1.00	0.93		1.00	0.96	
T-Test with 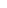 -adjustment:	1.00	1.00		1.00	1.00	
Random Forest:	0.50	1.00		0.30	1.00	
**b) semi-informative variables**						
New Method:	1.00	0.87	12; 10	1.00	0.81	11
T-Test without 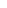 -adjustment:	1.00	0.96		1.00	0.95	
T-Test with 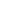 -adjustment:	0.00	1.00		0.50	1.00	
Random Forest:	0.70	0.99		0.30	1.00	
New Method:	1.00	0.74	14; 16	1.00	0.67	15
T-Test without 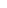 -adjustment:	1.00	0.96		1.00	0.95	
T-Test with 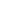 -adjustment:	0.70	1.00		0.60	1.00	
Random Forest:	1.00	0.90		0.30	1.00	
New Method:	1.00	0.59	30; 30	1.00	0.39	30
T-Test without 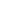 -adjustment:	1.00	0.95		1.00	0.96	
T-Test with 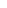 -adjustment:	0.90	1.00		1.00	1.00	
Random Forest:	1.00	0.98		1.00	0.92	
**c) non-informative variables**						
New Method:	0.10	0.87	12; 10	0.10	0.81	11
T-Test without 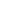 -adjustment:	0.00	0.96		0.00	0.95	
T-Test with 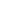 -adjustment:	0.00	1.00		0.00	1.00	
Random Forest:	0.00	0.98		0.00	1.00	
New Method:	0.40	0.75	14; 16	0.30	0.69	15
T-Test without 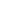 -adjustment:	0.00	0.95		0.00	0.95	
T-Test with 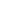 -adjustment:	0.00	1.00		0.00	1.00	
Random Forest:	0.10	0.97		0.00	1.00	
New Method:	0.40	0.57	30; 30	0.70	0.40	30
T-Test without 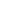 -adjustment:	0.10	0.95		0.00	0.96	
T-Test with 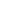 -adjustment:	0.00	1.00		0.00	1.00	
Random Forest:	0.00	1.00		0.00	1.00	

Validity of the measure of relevance for the two sample-problem evaluated by sensitivity and specificity. For comparisons we used the a multiple-testing-adjusted approach based on the t-test and the tree-based Random Forest approach. For informative or semi-informative metric data sensitivity showed good concordance with multiple testing without 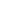-adjustment. This was irrespective of sample design and sample size. Specificity did not show as good results as sensitivity (for more details see text). However, the specificity results were better than those obtained from t-test without multiple-testing-adjustment.

Among 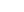 simulation runs, each with normal-distributed pseudo random numbers for 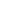 informative and 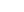 non-informative variables, it is also possible to obtain some large effects (normalized biological differences) by random among the non-informative variables. With increasing sample sizes the stop criterion always becomes smaller, so the risk for obtaining more non-informative variables with 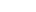-values larger than the stop criterion will increase with increasing sample size. They will then be falsely identified as informative which explains the weak specificity of the measure of relevance for large samples. In practice, however, is not advisable to declare variables with small differences in their group means automatically as non-informative. Whenever such variables posses a negligible variance in the two groups as well, they will point to large effects and therefore, should be declared as informative even if their group mean differences are small.

**Figure 3:**
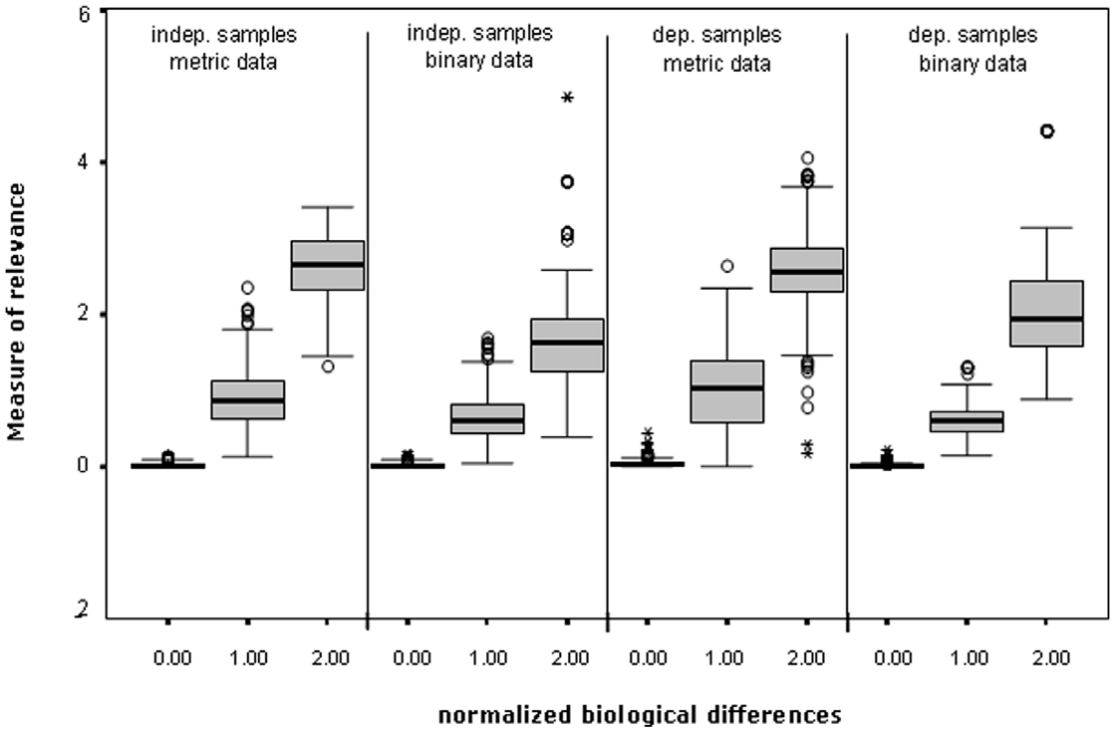
Boxplots of the MoR values under diverse sample designs and data structures. Irrespective of data type and sample design in the two-sample problem, the measure of relevance shows similar values if the variables are very informative (Normalized Biological Difference (NBD) about 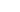), semi-informative (NBD about 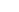) or non-informative (NBD about 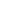).

We additionally performed a simulation study to investigate whether the 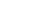 values could be compared, if different data structures and sample designs are used. Figure (3) depicts Box-graphs showing the distribution of the measure of relevance based on these simulations.

The sample size was constant in all samples and all simulations 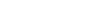. The information content was assigned not only to 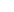 variables but to all considered variables. For independent samples with metric data the simulated data for one sample was derived from 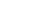, whereas for the other sample it was derived from 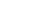 with 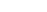, 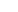 and 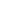 in order to produce normalized effect sizes equal to 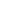 (non-informative), 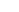 (semi-informative) and 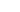 (informative), respectively. For dependent samples the simulations were similar to the case with independent samples, except for a correlation aspect which has been considered when choosing data for the second sample. Since comparing the 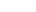-values of different data types (e.g. metric vs binary) is only meaningful when the data of different types contain almost similar information, we created the binary data for both independent and dependent samples as follows: The metric data of the 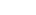 variables were transformed in binary data by giving the values 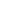 and 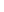 when data of the two samples were less or greater the whole-sample median, respectively. Interestingly, the box graphs corresponding to the same information contents, except a few outliers (extremes), do not show any denotative differences irrespective of data structure and sample design. We can therefore claim that the measure of significance could be applied in all data and sample situations without constraints.

### (d) Quality and practicability of the objective criteria

Finally, the simulation data and samples used for proving reliability were also employed to evaluate quality and practicability of the objective criteria for variable selection across the information chain. As objective criteria we considered those based on entropy, permutation and on the sample related cut-off formula. Independent on sample sizes, sample designs and distribution forms of the data, the three criteria selected the same variables as relevant, whenever they are indeed informative and differ substantially from the non-informative variables. Permutation-based criterion and the sample related cut-off criterion supply also similar results in cases of semi-informative variables. However, for semi-informative variables the entropy-based criterion shows some deviations from the others criteria, but this is understandable. If the 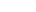 of the considered variables are very close across the information chain the entropy change between neighbor variables will be almost stable irrespective of the 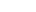 amounts. In such cases, 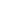 in inequalition 11 decreases monotonously and with almost equal decrements between adjacent 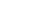
s and is after certain steps less than the chosen 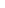. The smaller the chosen 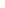 the more steps will the algorithm need to stop.

As an orientation in handling with the objective criteria for variable selection with the 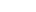 method is to prevail the following: The entropy-based criterion can be applied to any sample amounts. It works better than the other two criteria in cases of very small samples 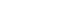. Values for 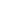 between 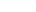 and 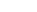 seem to be here very good. By large samples also the sample related cut-off criterion works very well and should be preferred because of its application simplicity. By moderate samples (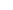,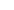 between 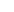 and 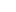) the permutation-based criterion delivers also acceptable results, but its application is very cumbersome and computationally intensive. Generally, the establishment of an unequivocal optimal stop criterion constitutes a further challenge and an interesting task for the future time.

### Some comments to more complex designs

The essence of the above explanation was the two sample-situation problem or, in other words, the multivariate problem with a single influential factor of two levels only. However, when studying complex phenomena in basic research, we seldom have to deal with such simple situations. The designs of basic research studies are generally more complex. They exhibit more than one influential factor, some of which may have more than two levels. Therefore, the following questions have to be addressed: [(1)]

1. How can we apply the measure of relevance in more complex situations (more influential factors or more than two levels)?
2. How can we study interaction effects of two or more influential factors on multivariate data?
3. Is there a possibility to investigate factor effects on interaction of biomarkers?

ad 1. For experimental designs incorporating one influential factor with more than two levels, we consider all possible pair combinations of these levels. With the 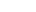 method we identify the most informative variables (biomarker candidates) for all level pairs. Since each level pair represents a two-sample situation, the identification of the biomarker candidates uses exactly the same procedure presented for the two-sample situation. However, the biomarkers identified under the various level pairs do not have to be identical and would probably differ in their amounts and signs. Depending on the focus of our interest we can thereafter, either use those identified by a certain level pair or declare all different biomarkers identified by the various level pairs as overall biomarker candidates, i.e. as variables characterizing the impact of the influential factor.

ad 2. The investigation of factor interaction in the case of two or more influential factors can be done only when the sample sizes are large. The necessary size of a population sample in such cases depends on the number of factors considered and their levels. Assuming, for example, a minimum of 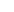 items per sample for the application of 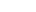 in the two-samples situation; then for factors with more levels it is recommended to use population samples equal or larger than 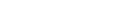, where 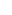 is the number of levels for the factor 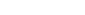). The former method is similar to that used in question (1).

ad 3. This question is more complicated and it addresses difficult numerical methods. In multivariate problems with, say 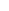 variables, the number 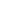 of all possible variable interactions is 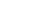. For large 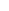 (e.g. 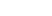), 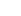 becomes very large and numerical operations and algorithms towards 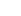 can not be computed easily. On the other hand, one has to ask, whether and which interactions between biomarkers make sense. Generally, scientists are not too interested in examining on which interactions of variables the influential factors exercise significant effects. They want, more or less, to investigate on which variables some preconceived influential factors and their interaction have a crucial influence. This point of view brings us back to questions (1) and (2). When, in high dimension data, interactions make sense, we recommend investigation of interaction/correlation effects in the last stage of the analysis, namely, after identifying the most relevant biomarkers by means of 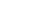. This method should be followed under certain conditions, also in the statistical inference based on multiple testing (see [11]).

### Examples

The following three examples demonstrate the power of this novel method in identifying relevant biomarkers.

#### 1) Sleep data example

Sleep is a very important component of life. Therefore, it is of immense relevance to know the physiology, structure and functional mechanisms of the sleep process as well the causes of its disturbances. In sleep research polysomnographic recordings including, electroencephalograms (EEG), electrocardiograms (ECG) and muscle activity (EMG), are commonly used. From these recordings a variety of variables (parameters) are extracted for detailed sleep data analysis. The resulting parameters may be classified in architecture, continuity or quantitative parameters representing respectively durations in the sleep states or transitions between sleep states or rhythmic intensities of certain frequencies and frequency bands as well.

The objective of a recent study ([12]) was to examine the effect of sleep deprivation on sleep and the secretion of a specific hormone (Renin) during the recovering night. A sample of 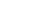 subjects investigated two times (before and after sleep deprivation) serves as background for the study.

The deprivation study is equivalent to a multivariate two situation problem with dependent samples and different data types. Therefore, the question ‘which sleep parameters show significant differences between nights before and after sleep deprivation’ is investigated by our method based on the measure of relevance. The results are depicted in figure (2b). The graph represents the ordered 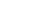 values and visualizes the magnitude of change of the considered sleep parameters after sleep deprivation. On the right side of the diagram are listed those variables which were identified as most relevant by the sample-related objective cut-off criterion, which in this case equals 
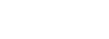
. Interestingly, in this study the measure of relevance revealed a lot of significant sleep parameters (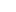 among 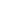), which appear to be too much. Nevertheless, we are not surprised by this result because sleep deprivation provokes general changes at many of the considered parameters ([12]). With the 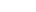 approach we endorse again that sleep deprivation is the most powerful method to promote sleep. During the recovery night following sleep deprivation sleep propensity is enhanced resulting in by the architecture parameters (AP) significant increases of slow wave sleep (SWS), rapid eye movement (REM), sleep-efficiency index (SEI)and sleep stage 3 and decreases of shallow sleep (sleep stage 1) and wakefulness. We found also significant increases after sleep deprivation in some continuity parameters (CP) like the transition frequencies from wake to light sleep and from sleep stage 2 to REM sleep as well as significant decreases in the transitions light sleep to wake. By the quantitative parameters (QP) the rhythmic intensities of Delta bands (Delta power) showed during the recovery night significant higher values than in the baseline. We can say that almost all old findings have also been found by 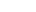 without any requirements of data structure, sample size and hypothesis testing which emphasizes the usefulness of the 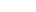 method in sleep research. Applying the non-parametric sign-test to variables that were detected as informative by the 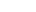, yielded p-values less or equal to the Bonferroni-adjusted 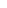. Therefore, we can assert here that the sleep parameters identified as the most relevant by 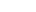 are the best biomarkers of sleep deprivation.

#### 2) An example with molecular-biology data

In this example we use data of a published study ([13]). The objective was to investigate which genes or gene mutations are responsible for panic disorder (PD), a disease with a lifetime prevalence up to 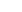 worldwide. To approximate this aim, genome-wide case-control association analysis based on about 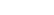 SNPs (single nucleotide polymorphism) across the entire genome were conducted. The study consisted of three stages. In each stage different samples of the patient- and control-populations were included in order to consider various aspects of the disease and to verify some findings. In the first stage, which is of particular interest for the scope of this paper, case-control association analysis were performed on 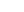 PD-patients and 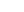 controls (discovery sample). The identified biomarkers were then used in further stages for identifying and verifying the most significant among them.

**Figure 4:**
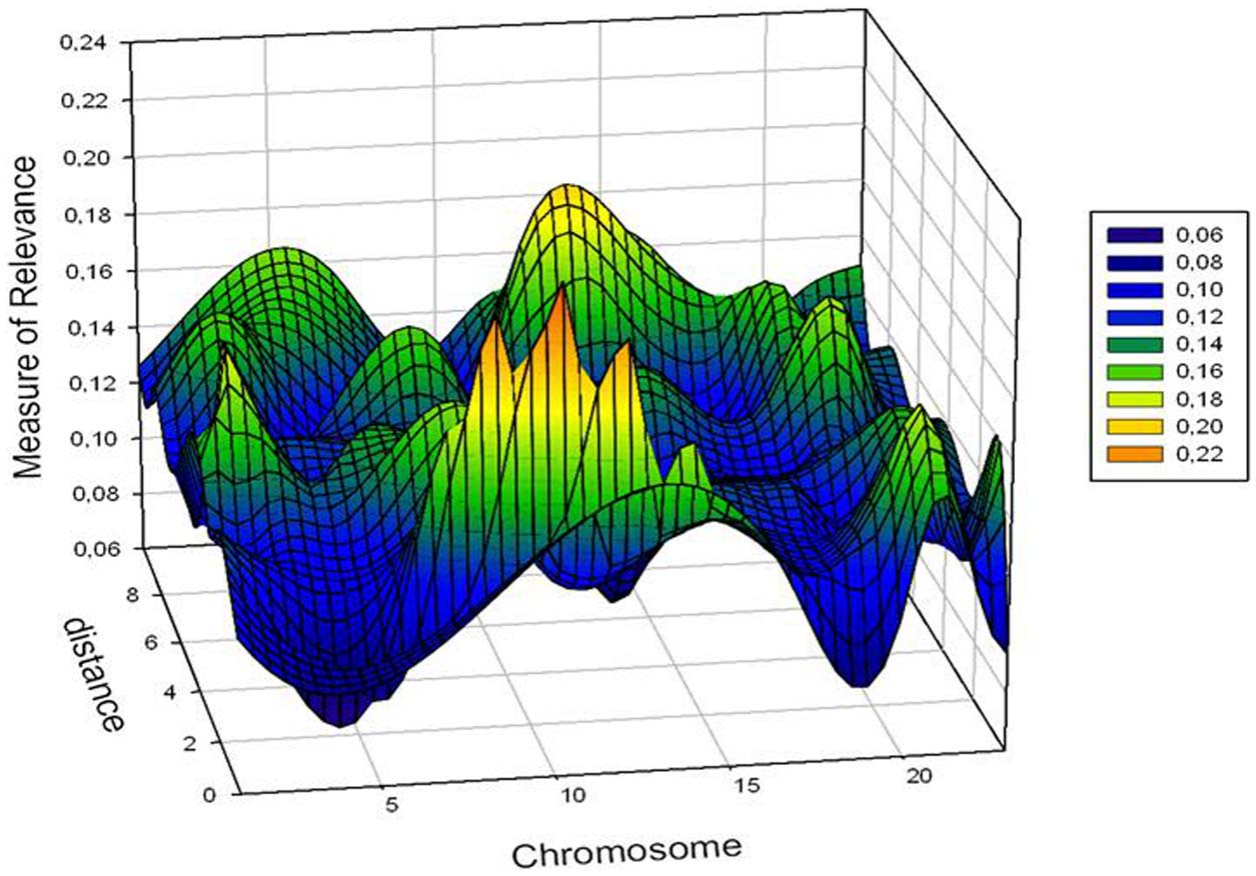
Mountain-valley view of the MoR-values corresponding to the most informative SNPs. Identifying genes with relevant differences in microarray-based expressions between HAB, NAB and LAB mice. Using the MoR method with the entropy-based stop criterion for variable selection 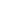, 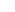 and 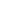 among 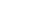 probes were proven to be very informative (relevant) in their expression profiles between HAB and NAB (black symbols over the black solid line), HAB and LAB (blue symbols over the blue solid line)and NAB and LAB (red symbols over the red solid line) mice, respectively. All relevant probes belong to the set of the 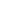 genes identified and declared with laborious methods from the study investigators as top candidate genes for further investigations.

The purpose of the study in each stage can be translated into the investigation of significant differences in a multivariate two-sample problem with independent samples and binary data structure. A common way to do this investigation is to calculate the 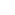-statistics for the allele-frequencies of the considered SNPs and then tested significance after correction for multiple testing. The investigators followed this method, but after correction none of the SNP remained significant in the first stage. They decided however to select the 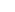 SNPs with the smallest p-values by the corresponding 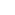-tests as biomarker candidates and to test their relevance in the other stages. In the second and third stages some SNPs that showed marginal significance at the corrected 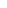-level in the first stage pointed to strong significant differences in their allele frequencies between PD patients and controls. The decision of the investigators to skip to the other stages proved to be wise. In the face of statistical inference, however, the changeover from the first to the other stages is a critical act. We decided to apply the 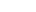 approach on a fraction of the data used in the first stage of the study. By randomly choosing two samples of 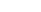 among the 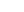 patients and 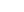 controls and calculating for each of the 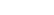 SNPs the corresponding measure of relevance we obtain, after sorting and application of the sample-related cut-off stop criterion with 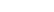, about 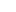 SNPs as biomarker candidates. With a few exceptions all the rest of these 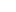 SNPs are including in the 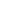 SNPs that showed marginal significance with the multiple-testing in the first stage of the study. By illustrating the relevance measures of the best 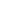 SNPs (see figure (4)) against chromosome and distance in 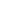 format (mountain-valley view) we can see, that some SNPs, especially on chromosomes 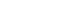 and 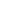, point to very large 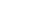
s and therefore seem to be the best biomarker candidates. Two of these SNPs are identical to those identified as significant in the other stages of the mentioned study. Therefore, if we had applied 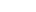 in the first stage of the study, the changeover to the other stages could take place without objection.

#### 3) An example with transcriptomic data

**Figure 5:**
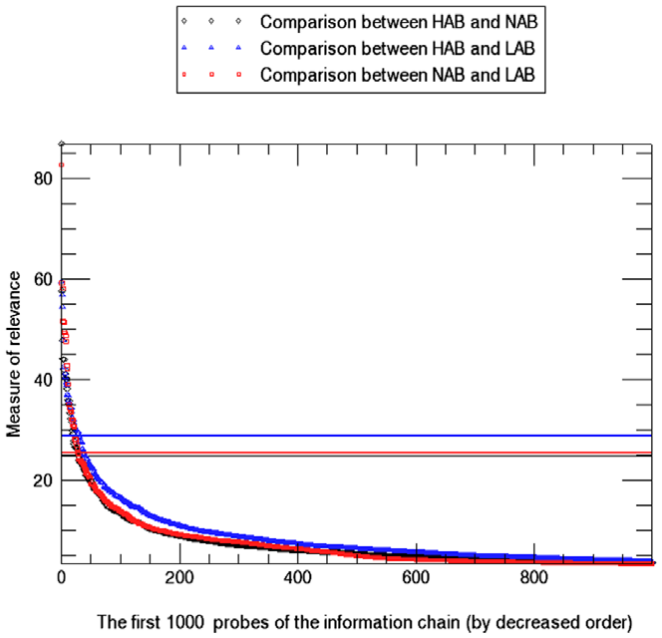
Measure of relevance for gene expression data. Application of the MoR approach to molecular biological data (SNPs) for identifying SNPs with relevant differences in the allele frequencies between controls and patients with panic disorder. In each group the SNP-data of 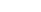 subjects were examined. For the two-independent-sample problem on hand with binary data the MoR-values of three SNPs on the chromosomes 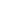, 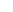 and 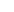 showed MoR-values above the sample-related cut-off criterion (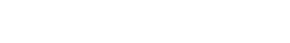).

To document the broad practicability of the 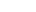 method, we provide an example on gene expression data of microarray analyses carried out on a small sample size (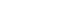). These data stem from a recently published work ([14]) carried out to investigate the effects of anxiety-related behavior on gene expression profiles. By using mice of three different bred lines characterized by high (HAB), normal (NAB) and low (LAB) anxiety behavior, the authors of the study used microarrays to investigate the gene expression profiles of different brain regions within the limbic system of these mice. For space reasons we focus our attention on the comparison of the gene expression data between the three lines in the gingulate cortex region only. Among the 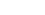 investigated probes, applying the 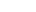 method on the normalized expression values with entropy-based criterion 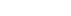 for variable selection we detected 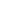, 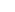 and 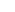 probes with very intensive (informative) regulation differences between HAB and NAB, HAB and LAB as well as NAB and LAB mice, respectively (see figure (5)). 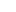 of these probes were common to all three comparisons. These 26 common probes correspond to genes being among the 32 top candidates genes selected by the authors under laborious statistical analyses for further investigations. Interestingly, all relevant probes recovered by 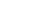 also belong to the 40 top candidate genes that have been detected by the authors after application of multiple testing and Bonferroni corrections on the 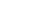 probes. Via the 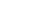 method we succeeded therefore in finding almost the same results as with inferential statistics. However, this time without testing and without any use of significance levels and corrections. This confirms the quality and application power of the 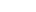 method for microarray analyses.

## Discussion

Modern digital and multimedia-based techniques enable an overabundant gain of information at almost any time and in almost any situation. In the past few years we have experienced an overflow of information and are astonished at how quick, precise and opulent this information is. Meanwhile, this trend is also found in research laboratories of almost all scientific disciplines. As stated in the introduction, any additional information about a phenomenon may contribute to a better explanation of it, but concurrently complicates the understanding and interpretation of its functions and mechanisms. For example, if the investigated phenomenon is a complex disease like major depression, the understanding of its genesis and synthesis requires abundant information on many levels (e.g. neuro-endocrinological, physiological, molecular-biological etc.). However, for therapy of the disease we want to know the most relevant influential factors, because the fewer the relevant factors, the better the treatment efficiency. Therefore, data shrinkage based on a reduction of dimension is the solution. But attention has to be paid so that loss of information is kept low.

One method of dimension reduction are the multidimensional scaling (MDS)-methods used in mapping and other visualization techniques (see [15]). Based on a distance matrix determined by a certain distance measures (e.g. the Euclid or Mahalanobis distance), multidimensional scaling is targeted to project 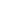-dimensional to 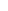-dimensional vectors (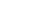) with minimal information loss. Factor analysis or principal component analysis are special applications of MDS-methods. One disadvantage of the MDS is, that components of the new vectors are expressed more or less as compounds of the components of the old vectors and can, therefore, be seldom identical with the original variables. Another problem is caused by the definition and use of distance measures. Since distance measures are determined over all vector components, object pairs often show the same distance values although their components are completely different. Hence MDS-techniques are not optimal for biomarker searching.

When the vector components are time-dependent or have the structure of a time series, a good possible method for variable reduction is functional fit over time. Polynomial or B-splines of a degree 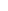 less than 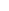 are applied to the time series and impose thereby a dimension reduction from 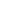 to 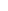. Functional fits show the same disadvantage as MDS, namely, the estimated components of the q-dimensional vectors are seldom identical to the original variables.

Another approach to achieve dimension reduction by time- or not time-dependent data may be obtained by the application of appropriate association models like generalized linear models, additive structured regression, functional regression, factor analysis, discriminant analysis, variance analysis etc (see e.g. [16]). In these models a response variable is usually associated with many other variables (predictors) and the objective is to explain the variability of it by the predictors. However, such models are only appropriate with adequate amounts of predictors, certain data structures and large samples. Whenever there are too many observed variables (many hundreds or many thousands) association models should not be applied without further ado. Beside the risk of collinearity, which would violate basic model assumptions, the shape of the functional form used is often questionable.

Also, some modern explorative methods such as Random Forest, LASSO, CART etc. which were developed for classification tasks, may determine the most relevant variables well (for details see [5]). However, the main principle behind these methods ‘the winner takes it all’ often metabolizes (especially by more than two classification possibilities) these methods in black boxes where the output is indeed relevant but not informative enough about the selected variables. ‘Identifying relevant predictor variables, rather than only predicting the response by means of some ‘black-box’, is of interest in many applications' ([17]). Moreover, in the face of too many variables the algorithms of these methods involve a risk of identifying some pseudo-correlations as true correlations. This fact may bias and distort the results. It is important to also mention here a weakness of methods that are based on split algorithms: Variables, which show in two-sample problems negligible differences between the two samples, will not be detected as relevant by methods based on split algorithms, irrespective of how small the variance is. This is undesirable for variables with small biological difference between two populations could be indeed of very large importance whenever they show small variance. Another disadvantage of those methods is that classification results obtained with a certain set of variables may be changed when additional variables have to be added to the set. Considering the sign of the 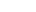, our new method enables, in contrast, not only the identification of relevant biomarkers, but also supplies information about the degree of relevance and the direction of the differences between the groups etc.

For high-throughput biomarker discovery some new strategies exist. They take into consideration possible correlations among the input variables. One example is the correlation modeling approach. It translates statistical inference to new data after modeling correlation structure with a special functional i.e. a spatial autoregressive model (see [18]). Another interesting approach uses new test-statistics, which combine the t-scores with estimated correlations (see [19]). A novel approach in association analysis is based on the correlation-adjusted t'-scores, which give optimal rankings in the t-scores when variables are correlated (see [20]). Nevertheless, all these methods work well with metric data but can not be transferred to other types of data without difficulties or strong compromises. We should again keep in mind that the investigation of group effects on variable interactions is not the same as searching for possible correlations between dependent variables in order to perform multiple testing more correctly. An interesting method for testing group effects on gene interactions (occasionally called epistatic interactions) is given by [21].

Our simulation studies revealed that the new method is at least as efficient as alternative approaches. However, in comparison to the alternatives, the new method for biomarker searching has some noteworthy advantages: First, it is free from association forms or functionals between response variable and predictors. Second, is free of classification risks, which are caused by pseudo-correlations in the case of high-dimensional data. Third, it operates on the explorative level and needs no correction of the level of significance for the identification of the best biomarkers. Fourth, it works well for any kind of experimental design and data. Last, but not least, it is very easy to apply.

It would be very interesting to investigate the robustness of 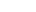 towards the weaknesses characterized by drawing multivariate samples in practice (e.g. poor representativeness, collinearity and dependency, outliers, mixed data structures, stratification, etc.). Of course the use of ranks provide an excellent basis for neutralizing outliers and different data-types, but as to whether it may also flatten some other troublesome effects is not clear. A good possibility for attenuating intrasampling weakness and simultaneously obtaining robust estimators and confidence intervals of the 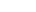 is provided by bootstrap sampling. Based on a random sampling, with replacement from the original sample, the desired number of resamples from the original data can be created. By calculating for each resample the 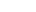 of the considered variables we can create for each variable as many 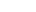
s as desired. Estimators and confidence intervals of the expected 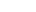 can then easy determined.

Nevertheless, by all advantages of 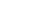 we must not forget, that it acts and operates on an explorative level. Therefore results obtaining by 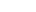 should be rather considered as trend-settings and not as ultima ratios. In the face of its comfortable applicability we plead to use 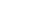 predominantly as an orientation and navigation tool in the prefields of basic research studies. After detecting with it the most relevat biomarker candidates study replications focused on all or part of these biomarkers are then recommended.

However, the method is still in progress. We are investigating the possibility to implement interactions, especially epistatic interactions of two, three and larger degrees. Also, we are trying to further optimize the selection criteria. Nevertheless, given that for each variable the relationship between the information content and amount of 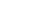 is unequivocal, the failure of an optimal selection criterion should not be considered as an essential impediment. By choosing from the (increasing) information chain the last 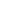 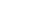
s, we should be sure that the corresponding variables are also the most informative. And that is enough for the start of a new method.

## Supporting Information

Appendix S1 Calculation of ***MoR*** for one variable
(PDF)
