# Supplementary material for: A New Way of Identifying Biomarkers in Biomedical Basic-Research Studies
Source: PLoS One. 2012 May 11;7(5):e35741. doi: 10.1371/journal.pone.0035741 (PMC3350515; doi:10.1371/journal.pone.0035741)
Supplement: Appendix S1 — Calculation of MoR for one variable (PDF) [file pone.0035741.s001.pdf]

Below are stated the mathematical formulas, that have to be used for the calculation of the measure of relevance by a simple variable only. In a two sample-problem let x_{i1}, x_{i2}, x_{i3} ,..., x_{in_i} $(i = 1, 2)$ denote the sample values of a stochastic variable X in the single samples. After attributing ranks to the sample values of the whole sample let u_{i1}, u_{i2}, u_{i3} ,..., u_{in_i}, $(i = 1, 2)$ indicate the corresponding transformed values of the ranks into the interval [0,1]. Thereby we use the formula $u_i = \frac{r_i - r_{min}}{r_{max} - r_{min}}$ for $i = 1, 2, ..., (n_1 + n_2)$. Based on the transformed data u_{ij} $(i = 1, 2; j = 1, 2, ..., n_i)$ the measure of relevance (MoR) should be calculated by the following formula:

$$MoR = \frac{1}{\sqrt{(n_1 + n_2)/2}} \cdot \frac{|p_1 - p_2|}{\sqrt{p \cdot (1 - p) \cdot (\frac{1}{n_1} + \frac{1}{n_2})}} \cdot \frac{(\bar{u}_1 - \bar{u}_2)}{\sqrt{s_1^2 + s_2^2 - 2 \cdot s_{12}}}$$

In the above formula, p_i, $i = 1, 2$ denote relative frequencies, $\bar{u}_i$ denote the means of transformed sample values u_{ij}, $i = 1, 2$, $j = 1, ..., n_i$ and s_1, s_2, s_{12} denote the standard deviations and covariance of the u_{ij}. p is defined as the ratio $\frac{n_1 p_1 + n_2 p_2}{n_1 + n_2}$ and equals to 0.5 for dependent samples.

For the determination of the parameters p_i, $i = 1, 2$ in formula (12) one has to differentiate between dependent and independent samples. Using the identity indicator $I(u)$ which takes 1 if z is true and 0 otherwise, p_i, $(i = 1, 2)$ is determined as follows:

$$p_{i,(i=1,2)} = \left\{ \begin{array}{ll} \sum_{i=1}^{n_i} \frac{1}{n_i} I(u_{ij} \geq \frac{n_1}{n_1 + n_2}), & \textbf{for independent samples} \\ \\ \sum_{i=1}^{n/2} \frac{1}{n/2} I(u_{1j} - u_{2j})\ inq\ 0, & \textbf{for dependent samples data} \\ & \text{(where } inq \text{ refers to '>' for } i = 1 \text{ and '<' for } i = 2) \end{array} \right\}$$
